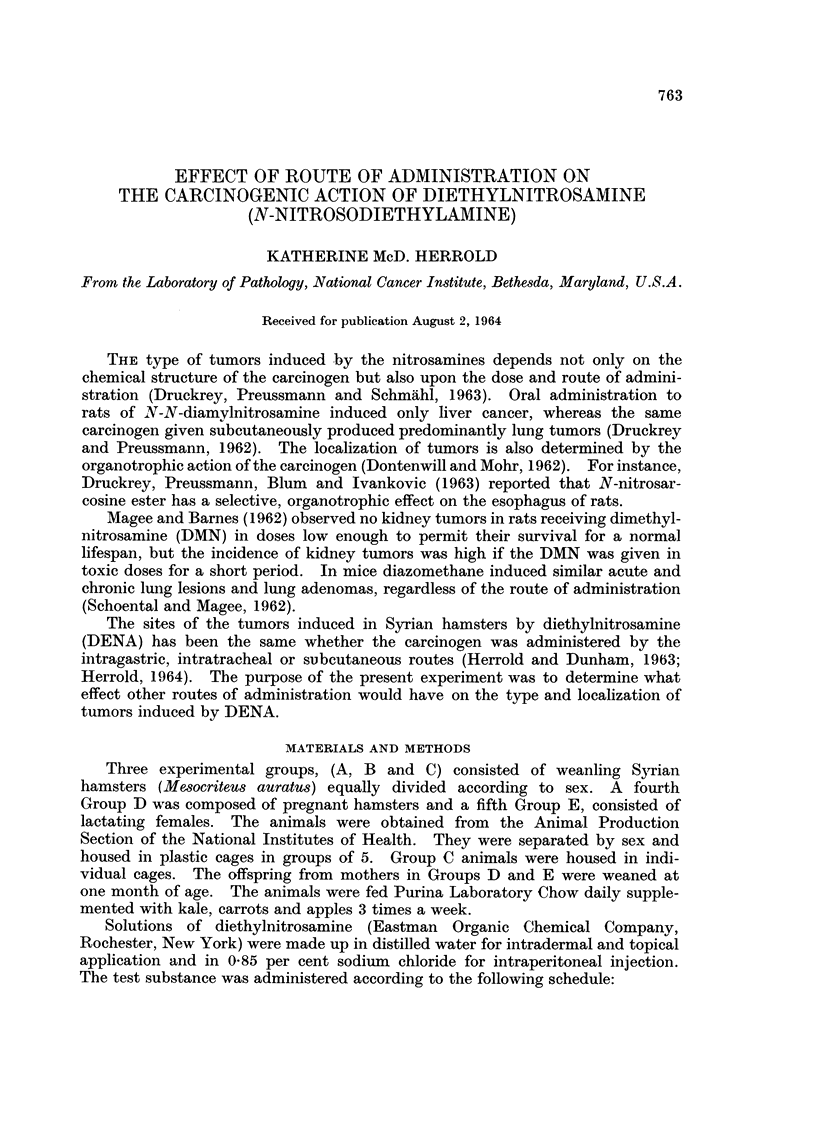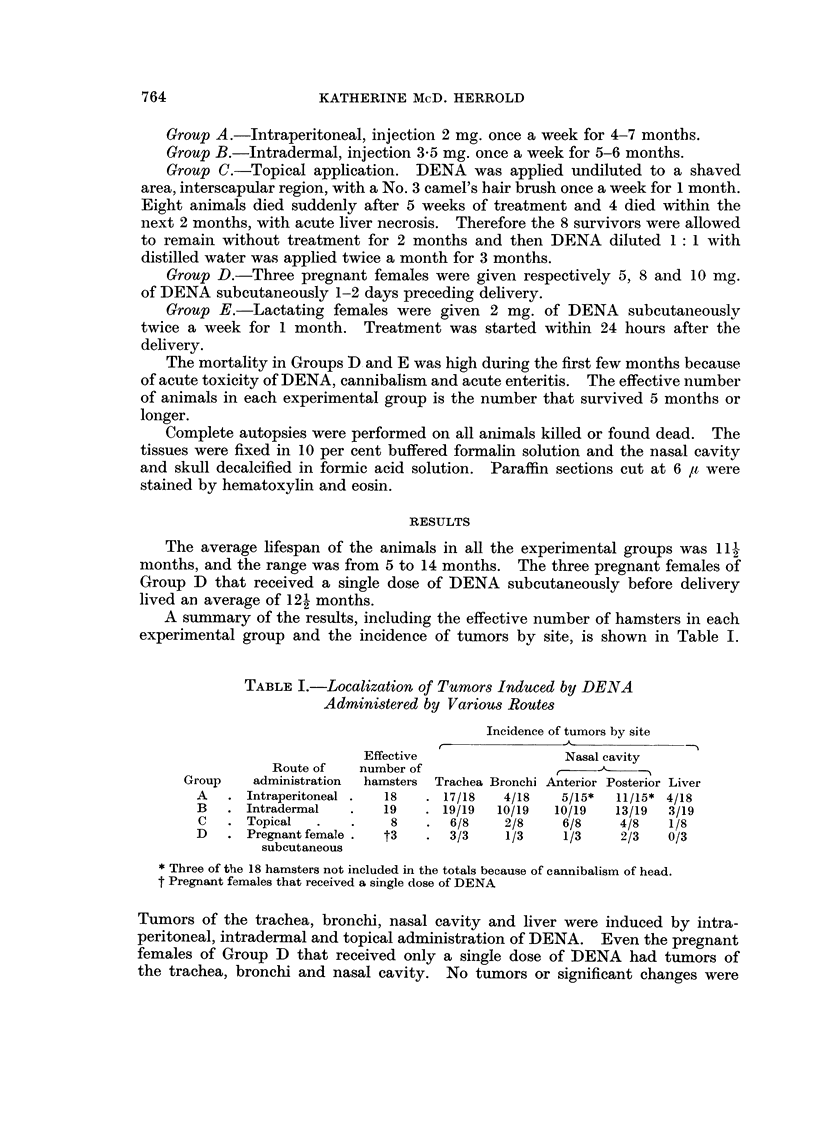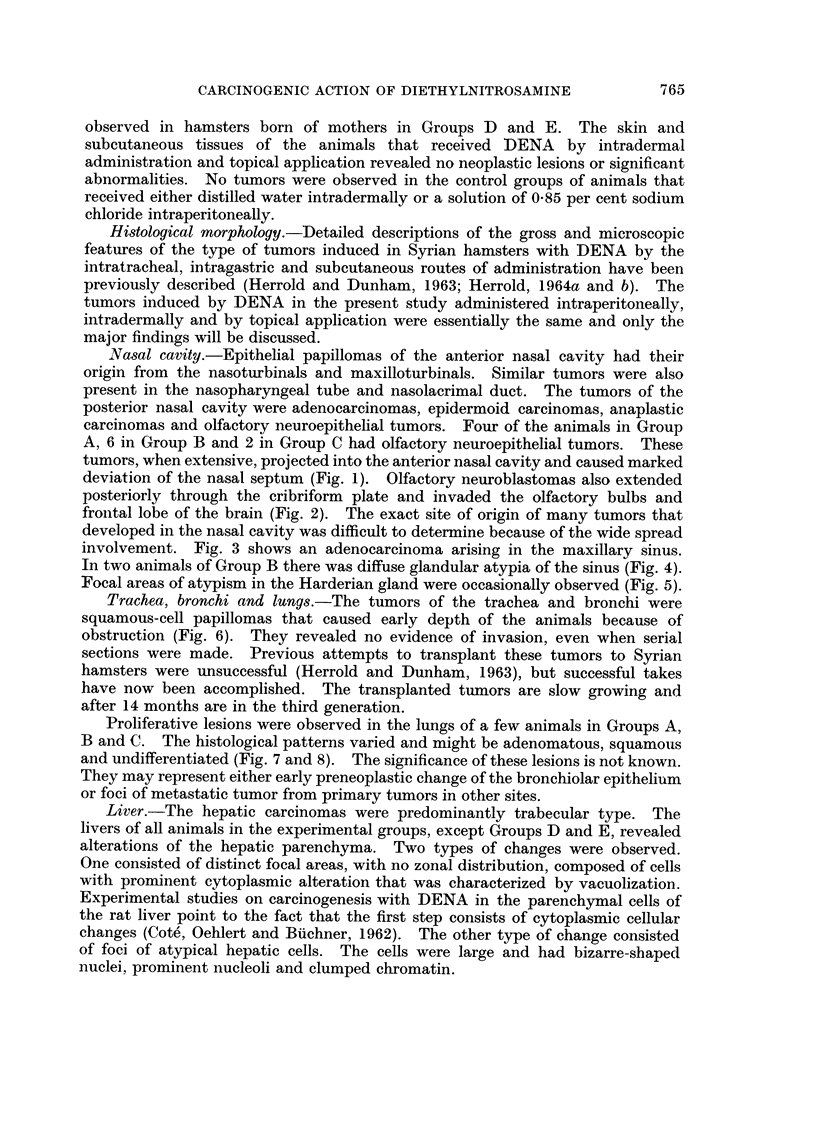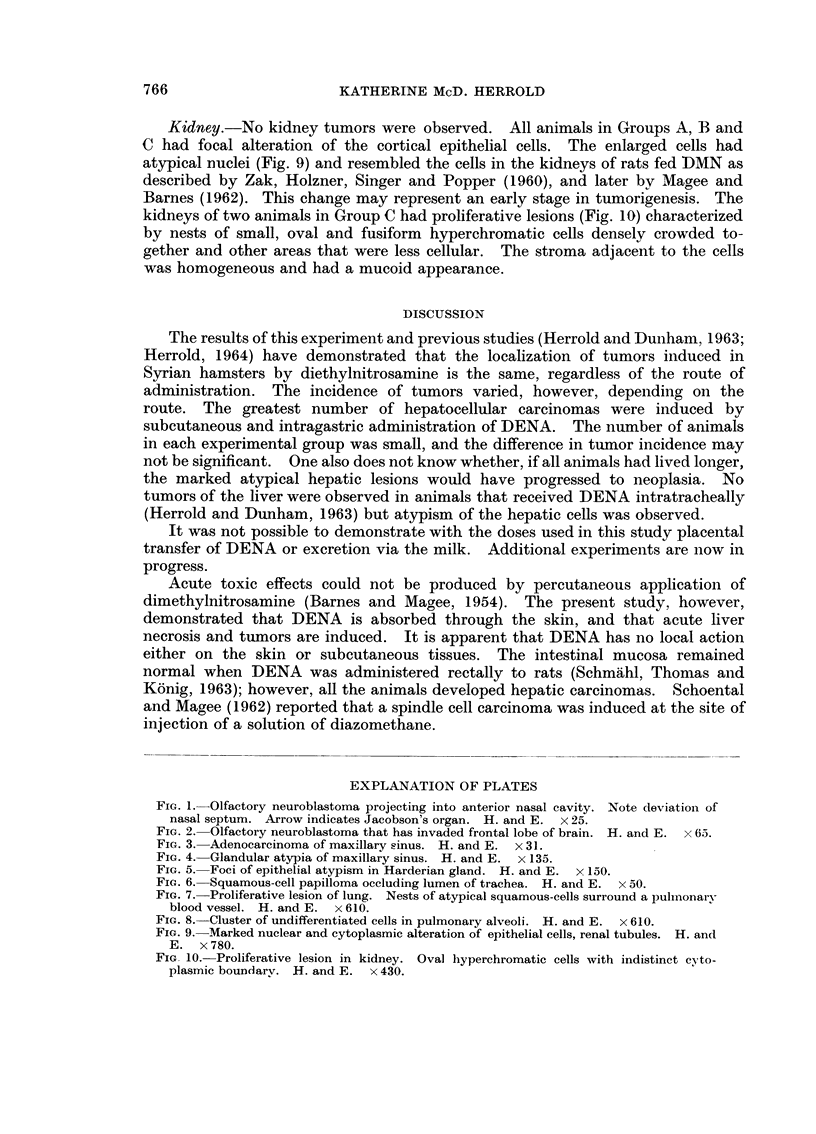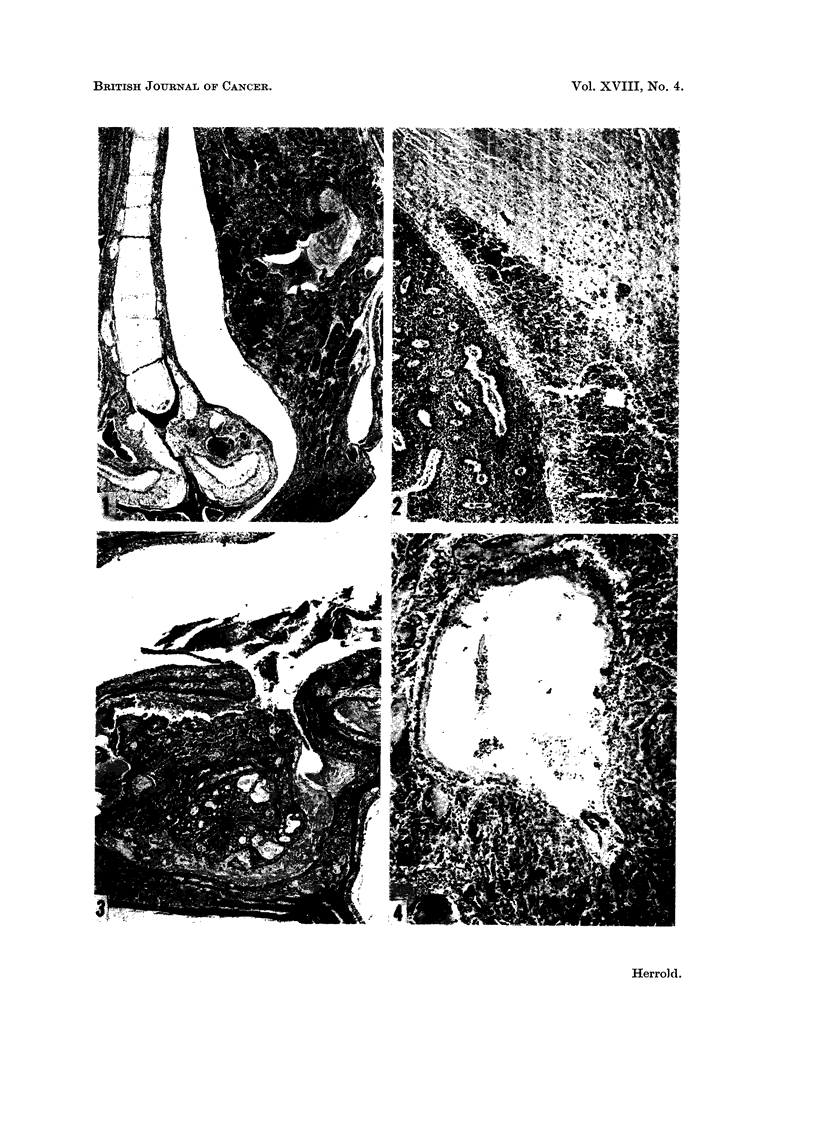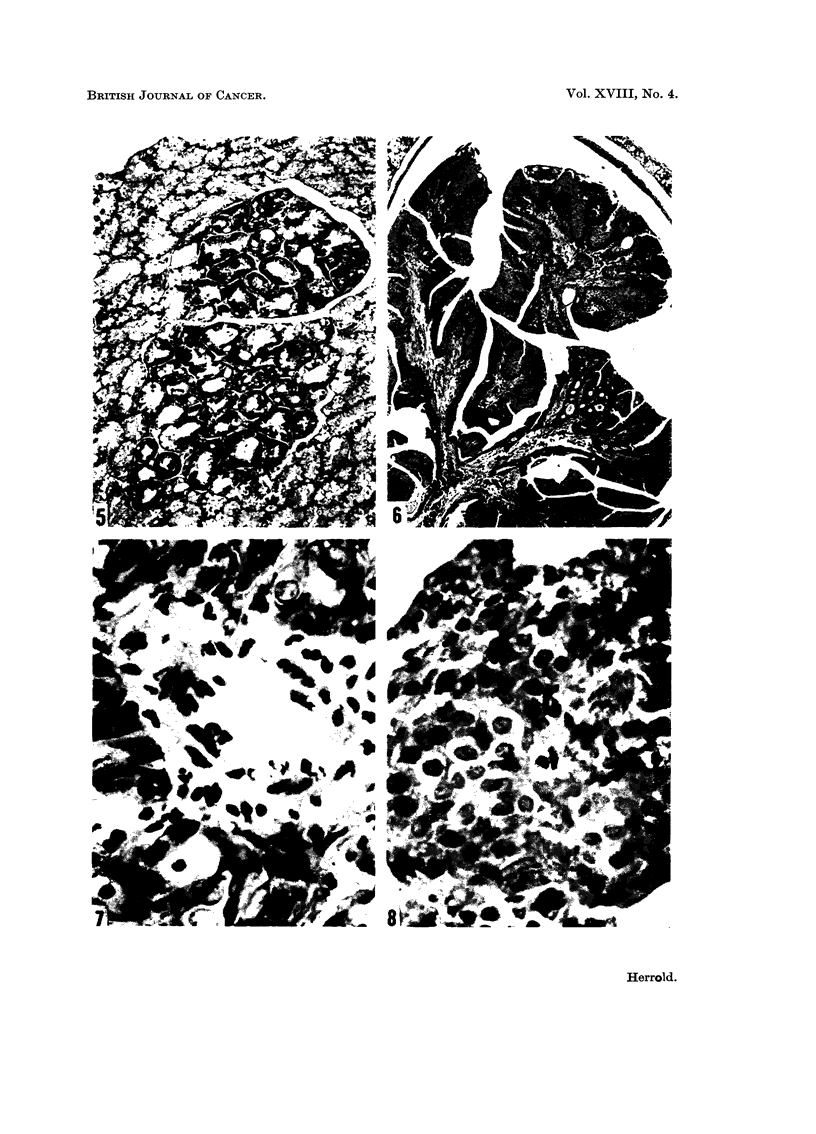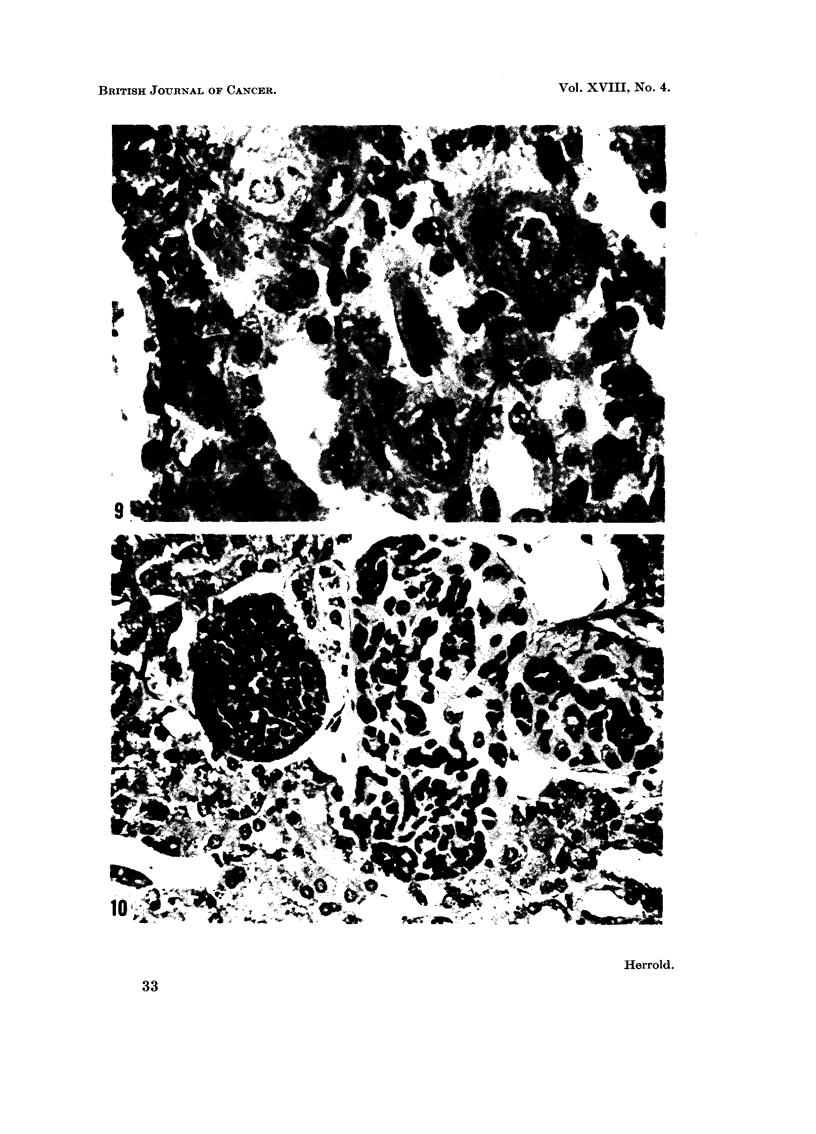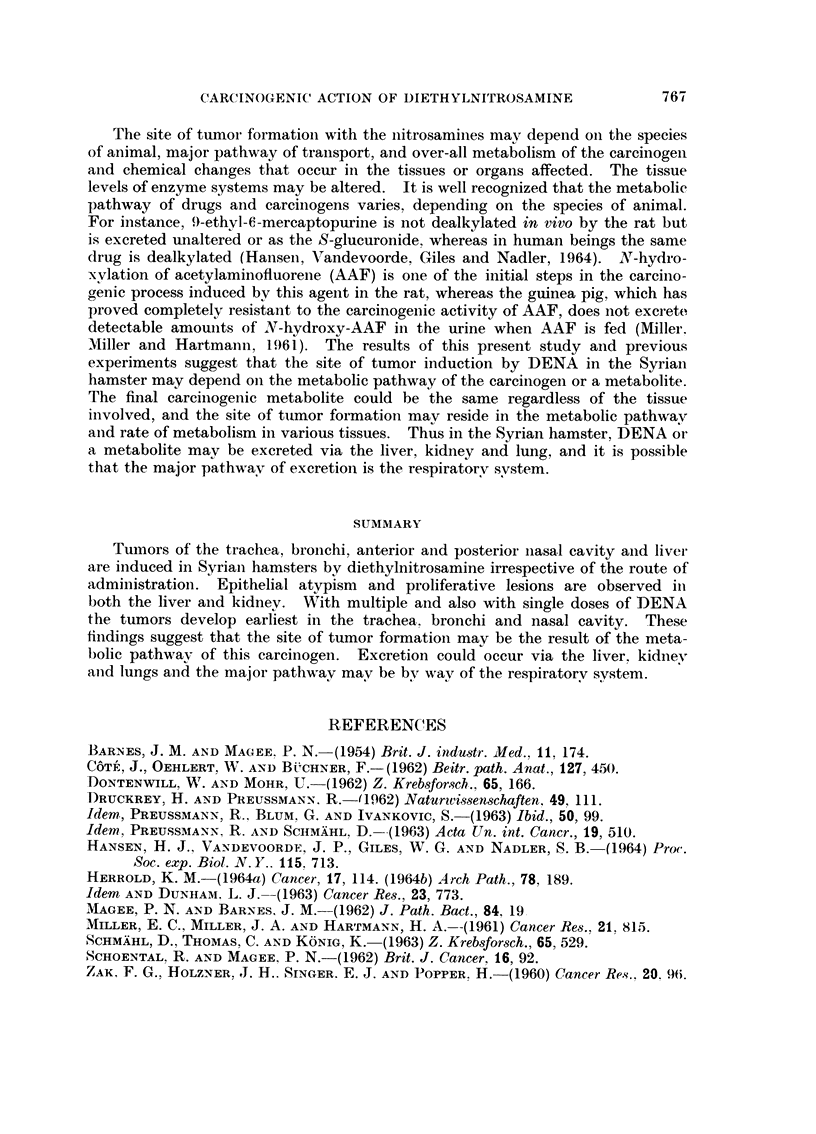# Effect of Route of Administration on the Carcinogenic Action of Diethylnitrosamine (N-Nitrosodiethylamine)

**DOI:** 10.1038/bjc.1964.88

**Published:** 1964-12

**Authors:** Katherine McD. Herrold

## Abstract

**Images:**


### EFFECT OF ROUTE OF ADMINISTRATION ON THE CARCINOGENIC ACTION OF DIETHYLNITROSAMINE (*N*-NITROSODIETHYLAMINE)

KATHERINE McD. HERROLD

*From the Laboratory of Pathology, National Cancer Institute, Bethesda, Maryland, U.S.A.*

Received for publication August 2, 1964

THE type of tumors induced by the nitrosamines depends not only on the chemical structure of the carcinogen but also upon the dose and route of administration (Druckrey, Preussmann and Schmähl, 1963). Oral administration to rats of *N*-*N*-diamylnitrosamine induced only liver cancer, whereas the same carcinogen given subcutaneously produced predominantly lung tumors (Druckrey and Preussmann, 1962). The localization of tumors is also determined by the organotrophic action of the carcinogen (Dontenwill and Mohr, 1962). For instance, Druckrey, Preussmann, Blum and Ivankovic (1963) reported that *N*-nitrosarcosine ester has a selective, organotrophic effect on the esophagus of rats.

Magee and Barnes (1962) observed no kidney tumors in rats receiving dimethylnitrosamine (DMN) in doses low enough to permit their survival for a normal lifespan, but the incidence of kidney tumors was high if the DMN was given in toxic doses for a short period. In mice diazomethane induced similar acute and chronic lung lesions and lung adenomas, regardless of the route of administration (Schoental and Magee, 1962).

The sites of the tumors induced in Syrian hamsters by diethylnitrosamine (DENA) has been the same whether the carcinogen was administered by the intragastric, intratracheal or subcutaneous routes (Herrold and Dunham, 1963; Herrold, 1964). The purpose of the present experiment was to determine what effect other routes of administration would have on the type and localization of tumors induced by DENA.

### MATERIALS AND METHODS

Three experimental groups, (A, B and C) consisted of weanling Syrian hamsters (*Mesocriteus auratus*) equally divided according to sex. A fourth Group D was composed of pregnant hamsters and a fifth Group E, consisted of lactating females. The animals were obtained from the Animal Production Section of the National Institutes of Health. They were separated by sex and housed in plastic cages in groups of 5. Group C animals were housed in individual cages. The offspring from mothers in Groups D and E were weaned at one month of age. The animals were fed Purina Laboratory Chow daily supplemented with kale, carrots and apples 3 times a week.

Solutions of diethylnitrosamine (Eastman Organic Chemical Company, Rochester, New York) were made up in distilled water for intradermal and topical application and in 0·85 per cent sodium chloride for intraperitoneal injection. The test substance was administered according to the following schedule:

*Group A.*—Intraperitoneal, injection 2 mg. once a week for 4–7 months.

*Group B.*—Intradermal, injection 3·5 mg. once a week for 5–6 months.

*Group C.*—Topical application. DENA was applied undiluted to a shaved area, interscapular region, with a No. 3 camel's hair brush once a week for 1 month. Eight animals died suddenly after 5 weeks of treatment and 4 died within the next 2 months, with acute liver necrosis. Therefore the 8 survivors were allowed to remain without treatment for 2 months and then DENA diluted 1 : 1 with distilled water was applied twice a month for 3 months.

*Group D.*—Three pregnant females were given respectively 5, 8 and 10 mg. of DENA subcutaneously 1–2 days preceding delivery.

*Group E.*—Lactating females were given 2 mg. of DENA subcutaneously twice a week for 1 month. Treatment was started within 24 hours after the delivery.

The mortality in Groups D and E was high during the first few months because of acute toxicity of DENA, cannibalism and acute enteritis. The effective number of animals in each experimental group is the number that survived 5 months or longer.

Complete autopsies were performed on all animals killed or found dead. The tissues were fixed in 10 per cent buffered formalin solution and the nasal cavity and skull decalcified in formic acid solution. Paraffin sections cut at 6 $\mu$ were stained by hematoxylin and eosin.

### RESULTS

The average lifespan of the animals in all the experimental groups was 11½ months, and the range was from 5 to 14 months. The three pregnant females of Group D that received a single dose of DENA subcutaneously before delivery lived an average of 12½ months.

A summary of the results, including the effective number of hamsters in each experimental group and the incidence of tumors by site, is shown in Table I.

TABLE I.—*Localization of Tumors Induced by DENA Administered by Various Routes*

| | | | Incidence of tumors by site | | | | |
|---|---|---|---|---|---|---|---|
| | | | | | Nasal cavity | | |
| Group | Route of administration | Effective number of hamsters | Trachea | Bronchi | Anterior | Posterior | Liver |
| A | Intraperitoneal | 18 | 17/18 | 4/18 | 5/15* | 11/15* | 4/18 |
| B | Intradermal | 19 | 19/19 | 10/19 | 10/19 | 13/19 | 3/19 |
| C | Topical | 8 | 6/8 | 2/8 | 6/8 | 4/8 | 1/8 |
| D | Pregnant female subcutaneous | †3 | 3/3 | 1/3 | 1/3 | 2/3 | 0/3 |

* Three of the 18 hamsters not included in the totals because of cannibalism of head.
† Pregnant females that received a single dose of DENA

Tumors of the trachea, bronchi, nasal cavity and liver were induced by intraperitoneal, intradermal and topical administration of DENA. Even the pregnant females of Group D that received only a single dose of DENA had tumors of the trachea, bronchi and nasal cavity. No tumors or significant changes were observed in hamsters born of mothers in Groups D and E. The skin and subcutaneous tissues of the animals that received DENA by intradermal administration and topical application revealed no neoplastic lesions or significant abnormalities. No tumors were observed in the control groups of animals that received either distilled water intradermally or a solution of 0·85 per cent sodium chloride intraperitoneally.

*Histological morphology.*—Detailed descriptions of the gross and microscopic features of the type of tumors induced in Syrian hamsters with DENA by the intratracheal, intragastric and subcutaneous routes of administration have been previously described (Herrold and Dunham, 1963; Herrold, 1964*a* and *b*). The tumors induced by DENA in the present study administered intraperitoneally, intradermally and by topical application were essentially the same and only the major findings will be discussed.

*Nasal cavity.*—Epithelial papillomas of the anterior nasal cavity had their origin from the nasoturbinals and maxilloturbinals. Similar tumors were also present in the nasopharyngeal tube and nasolacrimal duct. The tumors of the posterior nasal cavity were adenocarcinomas, epidermoid carcinomas, anaplastic carcinomas and olfactory neuroepithelial tumors. Four of the animals in Group A, 6 in Group B and 2 in Group C had olfactory neuroepithelial tumors. These tumors, when extensive, projected into the anterior nasal cavity and caused marked deviation of the nasal septum (Fig. 1). Olfactory neuroblastomas also extended posteriorly through the cribriform plate and invaded the olfactory bulbs and frontal lobe of the brain (Fig. 2). The exact site of origin of many tumors that developed in the nasal cavity was difficult to determine because of the wide spread involvement. Fig. 3 shows an adenocarcinoma arising in the maxillary sinus. In two animals of Group B there was diffuse glandular atypia of the sinus (Fig. 4). Focal areas of atypism in the Harderian gland were occasionally observed (Fig. 5).

*Trachea, bronchi and lungs.*—The tumors of the trachea and bronchi were squamous-cell papillomas that caused early depth of the animals because of obstruction (Fig. 6). They revealed no evidence of invasion, even when serial sections were made. Previous attempts to transplant these tumors to Syrian hamsters were unsuccessful (Herrold and Dunham, 1963), but successful takes have now been accomplished. The transplanted tumors are slow growing and after 14 months are in the third generation.

Proliferative lesions were observed in the lungs of a few animals in Groups A, B and C. The histological patterns varied and might be adenomatous, squamous and undifferentiated (Fig. 7 and 8). The significance of these lesions is not known. They may represent either early preneoplastic change of the bronchiolar epithelium or foci of metastatic tumor from primary tumors in other sites.

*Liver.*—The hepatic carcinomas were predominantly trabecular type. The livers of all animals in the experimental groups, except Groups D and E, revealed alterations of the hepatic parenchyma. Two types of changes were observed. One consisted of distinct focal areas, with no zonal distribution, composed of cells with prominent cytoplasmic alteration that was characterized by vacuolization. Experimental studies on carcinogenesis with DENA in the parenchymal cells of the rat liver point to the fact that the first step consists of cytoplasmic cellular changes (Coté, Oehlert and Büchner, 1962). The other type of change consisted of foci of atypical hepatic cells. The cells were large and had bizarre-shaped nuclei, prominent nucleoli and clumped chromatin.

*Kidney*.—No kidney tumors were observed. All animals in Groups A, B and C had focal alteration of the cortical epithelial cells. The enlarged cells had atypical nuclei (Fig. 9) and resembled the cells in the kidneys of rats fed DMN as described by Zak, Holzner, Singer and Popper (1960), and later by Magee and Barnes (1962). This change may represent an early stage in tumorigenesis. The kidneys of two animals in Group C had proliferative lesions (Fig. 10) characterized by nests of small, oval and fusiform hyperchromatic cells densely crowded together and other areas that were less cellular. The stroma adjacent to the cells was homogeneous and had a mucoid appearance.

### DISCUSSION

The results of this experiment and previous studies (Herrold and Dunham, 1963; Herrold, 1964) have demonstrated that the localization of tumors induced in Syrian hamsters by diethylnitrosamine is the same, regardless of the route of administration. The incidence of tumors varied, however, depending on the route. The greatest number of hepatocellular carcinomas were induced by subcutaneous and intragastric administration of DENA. The number of animals in each experimental group was small, and the difference in tumor incidence may not be significant. One also does not know whether, if all animals had lived longer, the marked atypical hepatic lesions would have progressed to neoplasia. No tumors of the liver were observed in animals that received DENA intratracheally (Herrold and Dunham, 1963) but atypism of the hepatic cells was observed.

It was not possible to demonstrate with the doses used in this study placental transfer of DENA or excretion via the milk. Additional experiments are now in progress.

Acute toxic effects could not be produced by percutaneous application of dimethylnitrosamine (Barnes and Magee, 1954). The present study, however, demonstrated that DENA is absorbed through the skin, and that acute liver necrosis and tumors are induced. It is apparent that DENA has no local action either on the skin or subcutaneous tissues. The intestinal mucosa remained normal when DENA was administered rectally to rats (Schmähl, Thomas and König, 1963); however, all the animals developed hepatic carcinomas. Schoental and Magee (1962) reported that a spindle cell carcinoma was induced at the site of injection of a solution of diazomethane.

---

### EXPLANATION OF PLATES

FIG. 1.—Olfactory neuroblastoma projecting into anterior nasal cavity. Note deviation of nasal septum. Arrow indicates Jacobson's organ. H. and E. ×25.

FIG. 2.—Olfactory neuroblastoma that has invaded frontal lobe of brain. H. and E. ×65.

FIG. 3.—Adenocarcinoma of maxillary sinus. H. and E. ×31.

FIG. 4.—Glandular atypia of maxillary sinus. H. and E. ×135.

FIG. 5.—Foci of epithelial atypism in Harderian gland. H. and E. ×150.

FIG. 6.—Squamous-cell papilloma occluding lumen of trachea. H. and E. ×50.

FIG. 7.—Proliferative lesion of lung. Nests of atypical squamous-cells surround a pulmonary blood vessel. H. and E. ×610.

FIG. 8.—Cluster of undifferentiated cells in pulmonary alveoli. H. and E. ×610.

FIG. 9.—Marked nuclear and cytoplasmic alteration of epithelial cells, renal tubules. H. and E. ×780.

FIG. 10.—Proliferative lesion in kidney. Oval hyperchromatic cells with indistinct cytoplasmic boundary. H. and E. ×430.

1

2

3

4

Herrold.

Herrold.

The site of tumor formation with the nitrosamines may depend on the species of animal, major pathway of transport, and over-all metabolism of the carcinogen and chemical changes that occur in the tissues or organs affected. The tissue levels of enzyme systems may be altered. It is well recognized that the metabolic pathway of drugs and carcinogens varies, depending on the species of animal. For instance, 9-ethyl-6-mercaptopurine is not dealkylated *in vivo* by the rat but is excreted unaltered or as the *S*-glucuronide, whereas in human beings the same drug is dealkylated (Hansen, Vandevoorde, Giles and Nadler, 1964). *N*-hydroxylation of acetylaminofluorene (AAF) is one of the initial steps in the carcinogenic process induced by this agent in the rat, whereas the guinea pig, which has proved completely resistant to the carcinogenic activity of AAF, does not excrete detectable amounts of *N*-hydroxy-AAF in the urine when AAF is fed (Miller, Miller and Hartmann, 1961). The results of this present study and previous experiments suggest that the site of tumor induction by DENA in the Syrian hamster may depend on the metabolic pathway of the carcinogen or a metabolite. The final carcinogenic metabolite could be the same regardless of the tissue involved, and the site of tumor formation may reside in the metabolic pathway and rate of metabolism in various tissues. Thus in the Syrian hamster, DENA or a metabolite may be excreted via the liver, kidney and lung, and it is possible that the major pathway of excretion is the respiratory system.

### SUMMARY

Tumors of the trachea, bronchi, anterior and posterior nasal cavity and liver are induced in Syrian hamsters by diethylnitrosamine irrespective of the route of administration. Epithelial atypism and proliferative lesions are observed in both the liver and kidney. With multiple and also with single doses of DENA the tumors develop earliest in the trachea, bronchi and nasal cavity. These findings suggest that the site of tumor formation may be the result of the metabolic pathway of this carcinogen. Excretion could occur via the liver, kidney and lungs and the major pathway may be by way of the respiratory system.

### REFERENCES

BARNES, J. M. AND MAGEE, P. N.—(1954) *Brit. J. industr. Med.*, **11**, 174.

CÔTÉ, J., OEHLERT, W. AND BÜCHNER, F.—(1962) *Beitr. path. Anat.*, **127**, 450.

DONTENWILL, W. AND MOHR, U.—(1962) *Z. Krebsforsch.*, **65**, 166.

DRUCKREY, H. AND PREUSSMANN, R.—(1962) *Naturwissenschaften*, **49**, 111.

*Idem*, PREUSSMANN, R., BLUM, G. AND IVANKOVIC, S.—(1963) *Ibid.*, **50**, 99.

*Idem*, PREUSSMANN, R. AND SCHMÄHL, D.—(1963) *Acta Un. int. Cancr.*, **19**, 510.

HANSEN, H. J., VANDEVOORDE, J. P., GILES, W. G. AND NADLER, S. B.—(1964) *Proc. Soc. exp. Biol. N.Y.*, **115**, 713.

HERROLD, K. M.—(1964*a*) *Cancer*, **17**, 114. (1964*b*) *Arch Path.*, **78**, 189.

*Idem* AND DUNHAM, L. J.—(1963) *Cancer Res.*, **23**, 773.

MAGEE, P. N. AND BARNES, J. M.—(1962) *J. Path. Bact.*, **84**, 19

MILLER, E. C., MILLER, J. A. AND HARTMANN, H. A.—(1961) *Cancer Res.*, **21**, 815.

SCHMÄHL, D., THOMAS, C. AND KÖNIG, K.—(1963) *Z. Krebsforsch.*, **65**, 529.

SCHOENTAL, R. AND MAGEE, P. N.—(1962) *Brit. J. Cancer*, **16**, 92.

ZAK, F. G., HOLZNER, J. H., SINGER, E. J. AND POPPER, H.—(1960) *Cancer Res.*, **20**, 96.